# Competition is not necessarily a barrier to community mobilisation among sex workers: an intervention planning assessment from Zimbabwe

**DOI:** 10.1186/s12889-015-2118-2

**Published:** 2015-08-16

**Authors:** Sibongile Mtetwa, Joanna Busza, Calum Davey, Ramona Wong-Gruenwald, Frances Cowan

**Affiliations:** Centre for Sexual Health, HIV and AIDS Research Zimbabwe, Harare, Zimbabwe; London School of Hygiene and Tropical Medicine, London, UK; Deutsche Gesellschaft für Internationale Zusammenarbeit (GIZ) HIV Prevention Project, Harare, Zimbabwe; Centre for Sexual Health & HIV Research, Research Department of Infection &Population Health, University College London, London, UK

## Abstract

**Background:**

Community mobilization among female sex workers (SWs) is recognized as an effective strategy to empower SWs and increase their uptake of health services. Activities focus on increasing social cohesion between SWs by building trust, strengthening networks, and encouraging shared efforts for mutual gain. Several studies, however, suggest that high levels of interpersonal competition between SWs can pose a barrier to collective action and support. We conducted a study to examine levels of perceived competition between SWs in Mutare, Hwange and Victoria Falls in Zimbabwe in order to inform development of a community-based intervention for HIV prevention and treatment. This paper focuses on our qualitative findings and their implications for the design of HIV programming in the Zimbabwean context.

**Methods:**

Following a respondent driven sampling (RDS) survey, we explored issues related to social cohesion amongst SWs in Mutare, Hwange and Victoria Falls through in-depth interviews conducted with 22 SWs. Interviews examined dynamics of SWs’ relationships and extent of social support, and were analyzed using thematic content analysis using the constant comparative method. Findings are contextualised against descriptive data extracted from the survey, which was analysed using Stata 12, adjusting for RDS.

**Results:**

Across all sites, women described protecting each other at night, advising each other about violent or non-paying clients, and paying fines for each other following arrest. In Mutare, women gave additional examples, including physically attacking problem clients, treatment adherence support and shared saving schemes. However, interviews also highlighted fierce competition between women and deep mistrust. This reflects the reported mix of competition and support from the survey of 836 women (Mutare *n* = 370, Hwange *n* = 237, Victoria Falls *n* = 229). In Mutare, 92.8 % of SWs agreed there was a lot of competition; 87.9 % reported that SWs support each other. This contrasted with Victoria Falls and Hwange where fewer agreed there was competition between SWs (70.5 % and 78.0 %), but also fewer reported that SWs support each other at work (55.2 % and 51.2 %).

**Conclusions:**

Women reported being most likely to support each other when confronted with serious danger but maintained high levels of competition for clients, suggesting competition at work does not represent a barrier to support. Examples of practical assistance between SWs provide entry points for our planned community mobilization activities, which aim to broaden trust and support among SWs while acknowledging their professional competition.

## Background

Sex workers (SWs) comprise one of the global key populations disproportionately affected by HIV. A recent systematic review found that HIV prevalence among female SWs ranges between 10–18 times higher than that of women of reproductive age in the general population [1]. In 2012, Zimbabwe’s national SWs HIV Prevention Programme reported 50 % HIV prevalence among SWs attending their clinics; the most recent UNAIDS country report also estimated 50 % prevalence among SWs, compared to 15 % among the general adult population [2]. These figures are consistent with HIV prevalence data for SWs throughout the region, for example, 59.6 % in South Africa, 70.7 % in Malawi, and 45.1 % in Kenya [1]. Provision of targeted, accessible and effective SWs prevention and treatment services thus remains urgent.

Community mobilization among female SWs has emerged as an effective HIV prevention strategy [3, 4]. The evidence suggests that by encouraging SWs to identify as part of a community with shared interests and concerns, and supporting collective action to address these concerns, community mobilization interventions can lead to greater risk reduction than behavioural and biomedical activities alone [5]. Community mobilization programmes focus on bringing women together for participatory activities to build trust, strengthen social networks, and enhance both individual and collective agency in addressing structural determinants of their vulnerability to HIV [6, 7]. Perhaps the best documented successes in community mobilization among female SWs come from India, where the well-renowned Sonagachi programme [8, 9] has been replicated and scaled-up across six states in India through the Avahan initiative [10, 11]. Avahan works to transform SWs’ role in health interventions from mere participants to social change agents with collective agency, ownership, and eventual sustainability of local organisations [12, 13]. Avahan has demonstrated positive outcomes including reduced vulnerability to violence [14, 15], improved relations with police [13], and consistent condom use positively associated with exposure to community mobilization interventions [16].

Not all programmes attempting to initiate community mobilization among SWs have reported success; several studies highlight how specific contextual factors can limit willingness to engage in collective activities or work toward mutual gain [4, 17, 18] Among these, a high level of interpersonal competition between SWs (or between the establishments where they work) has been identified as a significant barrier [19, 20]. Even within Avahan, some settings present greater challenges to the mobilization process than others. A qualitative study examining Avahan’s specific experience in Mumbai found that a much more challenging context for establishing an enabling environment for SWs’ collective action [11]. Greater control over SWs by brokers and pimps, and a fragmented, mobile and heterogeneous SW population weakened potential for women to come together and organise for mutual gain. Furthermore, many were reluctant to self-identify as SWs, one of the key first steps in the Avahan model [10].

Few examples of successful community mobilization come from African countries. A recent systematic review of sexual and reproductive health interventions for SWs in 26 African countries used Avahan’s conceptual framework to classify community empowerment into the four stages of (1) engagement (2) involvement (3) ownership and (4) sustainability, concluding that most of the 42 projects reviewed had not progressed beyond “engagement”, and there was insufficient evidence for supporting any given model for community mobilization in the region [21]. Early attempts to mobilize SWs in two different South African sites, a Johannesburg suburb [22] and a rural mining community [23], found SWs struggled to cooperate in enforcing condom use due to fierce competition over clients.

Understanding existing levels of mutual support, trust, and competition is thus critical to developing feasible SWs community mobilization activities relevant to the local context. We conducted a baseline assessment of support and competition among SWs in three Zimbabwean towns prior to adding community mobilisation activities to behavioural and clinical interventions to the National ‘Sisters with a Voice’ Programme. Our aim was to identify how SWs perceive relationships to peers in each site, with a focus on the extent to which they compete or collaborate on work and health related issues relevant to HIV programming. Results informed the design of participatory activities delivered as part of Zimbabwe’s national HIV prevention and treatment programme for SWs in these sites.

## Methods

This study was conducted in 2011 and included a respondent driven bio-behavioural survey and in-depth qualitative interviews with self-identified sex workers in Mutare, Hwange, Victoria Falls. Mutare is the third largest city in Zimbabwe, located close to the border with Mozambique as well as in the vicinity of gold and diamond mines, attracting migrant male workers in both transport and extraction industries. Both local and in-migrant women sell sex from hostels, truck stops, bars and clubs, and on the street; there is a greater diversity of prices and sex work typologies than in Hwange or Victoria Falls. Hwange is a small site dominated by a colliery and power station; SWs operate from bars and accommodation directly onsite as well as along the main road serving transport access for processed coal. Finally, Victoria Falls is a tourist town on the border with South Africa and Zambia, characterised by a high turnover of both local residents and visitors. Although sex work is considered more “high end,” taking place primarily in hotels and bars, prices have been lowered by the large number of women drawn to the site. Hwange and Victoria Falls are located within several hours drive of each other, and many SWs have experience of both locations.

We used respondent driven sampling (RDS) to determine SWs’ demographic and behavioural characteristics as well as explore local social contexts. We selected RDS due to the hidden nature of much sex work in Zimbabwe (particularly that outside of bars or hotels) and its demonstrated effectiveness in reaching diverse SW populations [13, 24–26].

A detailed description of the RDS methods used in the survey has been published elsewhere [27]. Briefly, following mapping of sex work venues, 22 seeds were selected to represent diversity by type of sex work, age, and location (10 in Mutare, 6 each in Victoria Falls and Hwange). Each was asked to recruit up to 2 other women and paid US$5 for completing the survey and a further US$2 for each of her recruits who also completed the process. In total, we recruited 836 SWs (Mutare *n* = 370, Hwange *n* = 237, Victoria Falls *n* = 229). Questions on SWs’ relationships with each other addressed mutual support, willingness to discuss issues related to work and health with peers, and nature and extent of competition; these were presented as statements using a 5-point Likert scale (“agree strongly,” “agree,” “neither agree nor disagree,” “disagree,” and “strongly disagree” or “very good,” “good” etc.). Data from the quantitative component were weighted to take account of RDS and analysed using Stata 12. Some quantitative findings are provided as background to each site to contextualise the qualitative data, on which this paper focuses.

We interviewed the 22 SW seeds (Mutare *n* = 10, Hwange *n* = 6, Victoria Falls *n* = 6) to further explore social support and community dynamics. As described above, seeds were selected based on their knowledge of the local SW community and involvement in a range of different venues (hostels, street, bars, home-based) and represented the range of ages and typologies of SWs working in these communities. Interviews probed the extent to which SWs looked out for each other, assisted each other in times of need, and encouraged each other to seek HIV-related services. Interviews were conducted by a female researcher from the sex worker HIV programme in participants’ chosen language (Shona or Ndebele) and compensated by US$5 for taking part as stipulated by Zimbabwean Medical Research Council guidelines. Interviews lasted roughly one hour and were digitally recorded, transcribed, and translated into English, with removal of all names and other personal identifiers. Transcripts were entered into N-Vivo 8 (QSR International Ltd, Melbourne, Australia) for thematic coding and content analysis using themes selected a priori*,* as the purpose of the qualitative study was to elicit greater detail on issues examined through the survey, particularly those found to affect community mobilisation efforts in other contexts.

Ethical approval for the study was obtained from the Medical Research Council of Zimbabwe (MRZC/A/1532), University College London and the London School of Hygiene and Tropical Medicine. All participants provided written informed consent.

## Results

The key survey findings related to social support and competition are shown in Table 1 to contextualise the interview data and provide background information that was available to guide our qualitative investigation. The median age of the women was 29 years old (range 18–67), and had worked longer in Mutare (median 5 years) than in Hwange (4 years) or Victoria Falls (3 years), with women reporting between from under one year to forty years of sex work experience.

**Table 1:** Quantitative social cohesion variables

	Hwange			Mutare			Victoria Falls		
	n	Un-weighted	Weighted % (95 % CI)	n	Un-weighted	Weighted % (95 % CI)	n	Un-weighted	Weighted % (95 % CI)
Total	237			370			229		
Median age		31			30			27	
Median years duration in sex work		4			5			3	
Median age started sex work		24			24			23	
% "good" or "very good"									
In general, how would you describe your relationships with other sex workers in this area?	147	62.0 %	65.7 (56.3 - 73.9)	237	64.1 %	62 (55–68.7)	143	62.4 %	61.4 (53.1 - 69.6)
% "strongly agree" or "agree"									
There a lot of competition between the women working here	189	79.7 %	78.0 (69.8 - 85.1)	338	91.4 %	92.8 (89.0 - 95.8)	166	72.5 %	70.5 (62.6 - 78.1)
Sex workers can work together to improve their condition	124*	93.9 %	94.6 (89.6 - 98.5)	313	84.6 %	85.4 (80.9 - 89.9)	130*	85.5 %	84.6 (74.5 - 93.6)
Sex workers support each other at work	117	49.4 %	51.2 (42.6 - 59.9)	331	89.5 %	87.9 (82.7 - 92.3)	117	51.1 %	55.2 (45.9 - 63.8)
My colleagues would help me if I refused to have sex with a client	113	47.7 %	54.0 (45.7 - 62.1)	292	78.9 %	79.8 (74.1 - 84.8)	97	42.4 %	43.5 (35.1 - 51.9)
My colleagues will help me if they see a client become aggressive or violent	215	90.7 %	88.9 (82.8 - 94.4)	359	97.0 %	96.9 (94.4 - 98.8)	218	95.2 %	95.0 (91.7 - 97.8)
I feel comfortable talking to other sex workers about work related issues	223	94.1 %	93.3 (89.3 - 97.2)	356	96.2 %	95.6 (92.0 - 98.3)	218	95.2 %	95.5 (92.3 - 98.2)
I feel comfortable talking to other sex workers about health topics	224	94.5 %	94.7 (91.2 - 98.1)	348	94.1 %	94.2 (91.3 - 96.7)	217	94.8 %	95.0 (91.8 - 97.8)
% " frequently" or "at least once a week" in the past month									
How often have you spoken to another sex worker about work?	149	62.9 %	58.1 (49.7 – 67.0)	195	52.7 %	44.9 (38.3 - 51.9)	126	55.0 %	53.6 (44.1 - 62.7)
% "yes"									
Did you ask another sex worker for help at work?	182	76.8 %	72.5 (63.7 - 80.4)	237	64.1 %	64.6 (58.5 - 70.5)	182	79.5 %	83.0 (75.1 - 90.3)
Did you provide help at work to another sex worker?	210	88.6 %	85.1 (77.7 - 91.4)	290	78.4 %	72.5 (65.6 –79.0)	202	88.2 %	88.6 (81.5 - 94.4)

Proportions for each variable are shown as un-weighted percentages and as RDS-weighted percentages with bootstrapped confidence intervals. The RDS weighting does not permit missing data or small group sizes, therefore un-weighted and weighted percentages are shown with women who refused to answer or did not know the answer in the denominator. In Hwange and Victoria Falls there was a large proportion of missing data for the question, Sex workers can work together to improve their condition”, with 32 women saying “don’t know” in Hwange, and 22 in Victoria Falls, as well as 73 “non-responses” in Hwange, and 55 in Victoria falls. By contrast, there was a total of 6 missing data points in Mutare. Therefore, for Hwange and Victoria Falls the proportions for this question do not include missing data in the denominator – the numerators are marked with an asterisk (*) and the dominators are 132 and 152, respectively. For other variables the amount of missing data at any one site was low, with an average of 1 and maximum of 7 data points missing per site. Therefore, they did not have important effects on the estimated proportion

Competition was reportedly highest in Mutare, where 92.8 % of survey respondents either Strongly agreed or Agreed with the statement “There is a lot of competition between women working here.” On the other hand, women in Mutare also reported the highest perception of work-related support, with 87.9 % believing that “SWs support each other at work” and 79.8 % agreeing that “my colleagues would help me if I refused to have sex with a client.”

Hwange and Victoria Falls reported lower rates of perceived competition between SWs at 78.0 % and 70.5 % respectively, but also reported lower work-related support at 51.2 % and 55.2 %. Expecting help for refusing a client hovered around half in each of these sites. Yet surprisingly, *experienced* levels of peer support appeared higher in Hwange and Victoria Falls than in Mutare. In Hwange, 72.5 % of SWs stated that they had asked another SW for help at work while 85.1 % reported having provided help at work. In Victoria Falls, 83.0 % requested help and 88.6 % provided help to a fellow SW. In Mutare, however, 64.6 % SWs reported asking for work related assistance while 72.5 % had provided it.

Overall, the majority of respondents (61–66 %) in all sites reported having “Very good” or “Good” relationships with other SWs. Similarly, almost all respondents agreed that “SWs can work together to improve their conditions”, with 94.6 % in Hwange, 84.6 % in Mutare and 85.4 % (95 % CI: 80.9 – 89.9) in Victoria Falls saying they Strongly agreed or Agreed. Finally, in all three sites over 90 % of SWs expressed feeling comfortable talking to their peers about work or health.

To delve into these contradictions and complexities, we analysed our qualitative transcripts by the themes of general competition and solidarity, support in the specific areas of work and health, and how SWs utilise their social networks for mutual gain.

### Competition & Cooperation

All interviewed SWs described high levels of professional competition, usually centred on getting the best paying clients.

*The issue has to do with clients. Conflict arises because people have taken each other’s clients. Let’s say you come here from Bulawayo and we see you taking our clients whilst we the owners of this place are watching. So that’s where the conflict comes from. (Hwange, age 21)*

The relative attractiveness of new SWs to clients (“a fresh face”) in a given location provoked jealousy from longer term residents, making it difficult for new SWs to join existing social networks. One respondent noted that “gatekeepers” protect local territory. Older SWs and those living long-term in the area were wary of newcomers.

*When you visit a new area, there are some gatekeepers in every area. So if they see new faces, it disturbs them. Sometimes they will always be behind you just to see what will be really going on. Sometimes they can even hire thieves [to rob] you. (Mutare, age 34)*

This competition for clients could culminate in violence and manipulation as SWs guarded their own “territories.”

*The relationship is not so good because sometimes we fight over clients. So this creates a lot of animosity. (Victoria Falls, age 33)*

This could lead to SWs’ guarding personal information carefully rather than sharing problems, to avoid secrets being used against them.

*Some may be jealous of you. Let’s say I tell her my secret that these days, my life is like this, I am on [ART] drugs. When she sees me in the beer hall with a client, she may be jealous of me and want the client too, and she can secretly tell the client that I am on drugs. The client will leave [me]. (Hwange, age 21)*

One woman believed SWs who successfully obtained clients did so using ‘juju’ (magic powers). This was one reason given for SWs’ refusing to support each other financially.

*There are some girls who use juju to attract men and you find that some may wait there all night while those who used juju will be getting all the clients. (Mutare, age 34)*

*… [I]n sex work, people don’t give each other money. The reason is that if I give someone my money, she can use juju on it and [then] I will not be able to make money. (Hwange, age 61)*

Yet the survey had highlighted that a majority of women considered themselves to have good relationship with their peers, and felt SWs could work together to improve their conditions. When asked how competition might affect relationships and mutual support, respondents suggested that while fighting between SWs was pervasive, it was often temporary and ultimately inconsequential.

*If you have a fight, one of us will then go to Mama and ask her to call the other part so that we can reconcile, if we fought, we ask then ask each other for forgiveness then we start again… (Mutare, age 24)*

Long term survival was seen to depend on the ability to put conflicts aside and assist one another on a daily basis. The importance of friendships came out strongly in interviews; friends were expected to pool resources, lend each other money, or “treat” each other to demonstrate their trust and cooperation.

*We fight but it will be for 2–3 days…We get into a cab as friends, in pairs, so if you are 4, you combine your money. If someone’s boyfriend has come to visit, we can get into his [car], when he has gone back, then we all go out at once. (Mutare, age 32)*

*Yes, it’s like you don’t get money every day, some days you get it and some days you don’t. If I get and refuse to share, then I will know that you won’t share your food. We must help each other, if I buy you beer using my money, I’m not supposed to demand the money because I would have bought for you but if I ask you to lend me money, then you would know that you will get your money back, but if it’s only buying each other, no one demands money, if you do that, it shows that you are no longer friends. (Mutare, age 24)*

### Work-related support

Women described diverse examples of work-related assistance, broadly falling into three categories: (1) ensuring each others’ safety, (2) contributing to emergency or unexpected costs, and (3) “checking in” on each other as surrogate family members.

*The client can be troublesome and you just hear some noise in their house…I will help her since she’s my next door neighbour…I go and knock on the door to find out what's going on… (Mutare, age 33)*

*Right now, there is a certain sex worker who passed away, we have buried her today, … so we contributed $10 per each sex worker to buy food [for the funeral]. (Hwange, age 36)*

*It’s only my friends who come (if I’m sick), they come and ask if I have eaten, they help me with anything that I want, and sometimes they cook for me (Victoria Falls, age 31)*

*We may stay far from each other, but we see each other every day, we check on each other daily. My friends are more important to me than my own relatives. (Mutare, age 45)*

Women also talked about encouraging each other to demand advance payment from clients, leaving money with others while going with a client to avoid being robbed, and coming to each others’ assistance when clients try to avoid payment.

### Health-related support

Almost all women across all three sites reported feeling comfortable talking to peers about health topics in the RDS. During interviews women spoke about encouraging each other to seek medical attention, and how they share relevant information among their colleagues and friends. Due to the high prevalence of HIV, several mentioned initiating and adhering to antiretroviral therapy (ART), although alternative sources of care for sexual and reproductive health problems were also mentioned.

*My friend is very helpful. Before you arrived, my friend called me and asked me to wake up and take my cotrimoxazole. She does that every day. Sometimes I forget to take them … she is always telling me to take them. (Hwange, age 28)*

*If someone gets sick with an STI, we can help each other, because amongst us some will be familiar with traditional herbs, we go and look for them and give [to] our friend…or take her there” (Mutare, age 40)*

As part of ensuring each other’s safety at work, SWs shared condom supplies and urged each other to use them, as well as warning others about particular clients with whom it was difficult to negotiate.

*That’s what we do with my friends, if we see a potential client, we ask each other’s opinion… maybe one of us knows that the person doesn’t want to use condoms, it will be a no… (Hwange, age 56)*

Health appeared to be the easiest topic for SWs to discuss and share experiences, although women who knew they were HIV positive were careful not to disclose their status to those who might tell potential clients in an effort to secure the business for themselves.

## Discussion

We examined levels of competition, cooperation, and networks of support among SWs in Zimbabwe in advance of initiating new community mobilization activities within an existing behavioural and biomedical HIV intervention. Following mixed results in our RDS survey, we conducted in-depth interviews to explore how competition and peer support co-existed at fairly high levels among SWs, finding that perception of good relations among SWs, belief in the potential to work for mutual gain, and widespread distrust and violent clashes did not appear contradictory to SWs but rather reflected the complex nature of their work and social networks. SWs highlighted that battles over clients, income, boyfriends, and territory were integral features of sex work, but did not hinder cooperative arrangements in other aspects of the job, such as vetting dangerous or difficult clients, working in pairs and coming to each other’s aid in the workplace.

Mutare provides the most striking illustration of this, as data showed near universal agreement that there was a lot of competition among SWs (over 90 %) yet also the highest rate of SWs’ believing that they could work together to improve their conditions. It is possible that in Mutare, a more enabling environment for cooperation has developed, leading to SWs’ expectation of receiving help and support when required. Although women in Hwange and Victoria Falls reported more direct experience of assistance in the previous month, this could indicate that such help has become taken for granted in Mutare and is no longer particularly noteworthy. A similar finding was reported in India, where comparison of the Avahan programme in Maharashtra and Tamil Nadu after 18 months suggested that pre-existing levels of collective identity affected intervention impact [18]. For example, in Tamil Nadu, where there had been few activities targeting SWs, the introduction of Avahan increased reported condom use by 21 % among participants, while Maharashtra, where there was a longer history of SW programmes, showed less dramatic change at 6.2 % condom use increase.

Within closely knit friendships, the SWs we interviewed described lending money, buying gifts, and “checking in” on each other. At the broader community level, respondents described contributing to funeral expenses, family emergencies and health costs for women in their wider peer group who were not necessarily close friends. These examples reflect a certain level of social cohesion, a concept often used to characterise the strength of a community’s “social fabric” and interconnectedness, which is further associated with positive health behaviours such as condom use [28]. Common features include mutual aid, trust, and support [29]. Our findings suggest that while mutual aid and support already exist among SWs, trust is still fragile and dependent on close friendships in an otherwise competitive and sometimes combative work environment.

Our interpretation of our data leads us to conclude that the foundations of social cohesion and support networks are in place across the three study sites, but it would be unrealistic to directly address competition between SWs at the early stages of our community mobilisation intervention. Both the survey and qualitative interviews highlighted the high prevalence of competition between SWs, but also its many negative outcomes; our key informants provided ample justification for why they avoid sharing personal information or risking premature intimacy in new friendships. Currently, no self-defined SW community exists to facilitate development of collective agency; in keeping with Avahan’s programme theory, therefore, SWs in our sites will require an extended period of “identification” to gain the confidence and skills required to progress to the stages of collectivisation and programme ownership [10].

As a result, we believe activities overly focused on breaking down mistrust and promoting solidarity across the population could backfire and jeopardise our broader mobilization efforts. Instead, we plan to focus on the “entry points” provided in SWs’ own accounts, such as SWs’ concern for each others’ safety in the work environment, ability to avoid dangerous clients and theft, assistance during times of personal need, and acknowledgment that some friendships become close enough for lending money or exchanging gifts and could eventually evolve into stronger social networks of support. As noted in Moore’s review, community mobilization programmes in Africa have shown potential, but may require additional time and effort to overcome the region’s particular socio-political constraints, including widespread heterogeneity in the industry, pervasive criminalisation, and weak state support structures [21].

Following this study, we have run regular community mobilization meetings in all 3 sites. Mutare, the largest town, has the highest attendance rates, with 50–100 women at every meeting compared to 10–30 women attending in Victoria Falls and Hwange. Meetings address a range of issues related to health, safety, social support, and legal rights and appear to positively affect the strength of networks among SWs. For example, a peer educator from Mutare recently described a “well known” code SWs have agreed to adopt to warn one another about violent or non paying clients. Our activities will continue to bring SWs together in shared social spaces and work to broaden opportunities for peer support, reinforce existing beliefs that SWs can work together, and encourage the community to find their own ways to challenge their competitiveness over time.

This study has several limitations, including small sample sizes for the qualitative research, making it difficult to identify contextual differences between the three sites, despite these being evident in the survey. Because interviews were conducted among the “seeds” for the RDS survey, and thus among women considered to be central to SWs’ social networks, findings could be biased toward a more positive depiction of social support and mutual assistance. Furthermore, validity of our RDS survey results relies on the method’s statistical assumptions and adjustments based on social network size, which are self-reported and thus impossible to verify.

## Conclusions

Despite several programmes showing interpersonal competition between SWs to be a barrier to mobilization of the SW community, our pre-intervention assessment found that SWs support each other in multiple ways, even in the face of fierce competition. Our study suggests that competition between SWs need not be mutually exclusive to the existence of functioning support networks. We intend to build on existing trust and cooperation between SWs and will use examples provided through the interviews as entry points for our community mobilization activities, with the aim of broadening the domains of SWs’ support while acknowledging the inherent competition in sex work.

## Abbreviations

ART: Antiretroviral therapy
HIV: Human immunodeficiency virus
RDS: Respondent driven sampling
SW: Sex worker
